# Dyspnea induced by hemidiaphragmatic paralysis after ultrasound-guided supraclavicular brachial plexus block in a morbidly obese patient

**DOI:** 10.1097/MD.0000000000028525

**Published:** 2022-01-14

**Authors:** Jiaxin Lang, Xulei Cui, Jia Zhang, Yuguang Huang

**Affiliations:** a Department of Anesthesiology, Peking Union Medical College Hospital, Chinese Academy of Medical Sciences and Peking Union Medical College, Beijing, China; b Department of Orthopedics, Peking Union Medical College Hospital, Chinese Academy of Medical Sciences and Peking Union Medical College, Beijing, China.

**Keywords:** brachial plexus block, case report, hemidiaphragmatic paralysis, obesity, phrenic nerve block

## Abstract

**Rationale:**

Hemidiaphragmatic paralysis (HDP) is a frequent complication of the brachial plexus block, caused by unintentional blockade of ipsilateral phrenic nerve. HDP did not rise enough alarm and attention to most anesthesiologists, because most patients with no coexisting comorbid diseases are asymptomatic and able to tolerate it. However, it may cause severe respiratory complication for patients with preexisting compromised cardiorespiratory function.

**Patient concerns:**

A 67-year-old woman with morbidly obesity was planned to receive opening reduction and internal fixation of right humeral shaft fracture under regional anesthesia considering less respiratory and cardiovascular system interference compared with general anesthesia.

**Diagnoses:**

After ultrasound guided supraclavicular brachial plexus block, the patient developed severe hypoxia and hypercapnia.Unintentional block of phrenic nerve and diaphragm paralysis was diagnosed by diaphragm ultrasound, which was considered as the main reason of severe hypoxia.

**Interventions:**

It led to a conversion from regional anesthesia to general anesthesia with endotracheal intubation for patient's safety and smooth operation.

**Outcomes:**

The unintentional phrenic nerve block leads to a prolonged ventilation time, length of stay in intensive care unit and length of stay in hospital.

**Lessons:**

This case report highlights the risk of diaphragm paralysis in morbidly obese patients. Though new diaphragm sparing brachial plexus block (BPB) methods were developed intended to reduce the risk of HDP, no approaches could absolutely spare phrenic nerve involvement. Therefore, clinicians should always consider the risk of HDP associated with BPBs. For each individual, a detailed preoperative evaluation and sufficient preparation are paramount to avoid serious complications.

## 1 Introduction

Brachial plexus block (BPB) is commonly used for upper extremity surgeries not only because of its optimum perioperative analgesia and stress response blocking effect, but also for its reported safety in relation to the cardiovascular and respiratory systems.^[1]^ Hemidiaphragmatic paralysis (HDP), caused by the unintentional blockade of the phrenic nerve, is a common complication of BPB. Anesthesiologists are aware of but not much concerned about HDP,^[2]^ since most patients with no coexisting diseases are asymptomatic and can tolerate it. However, subsequent phrenic nerve block may cause complications in some patients with pre-existing ventilation dysfunction, such as obese patients.

In recent years, obesity has become a major health problem worldwide. It is well established that obesity causes significant physiological changes in the airway, the respiratory and the cardiovascular systems. Thus, regional anesthesia is considered more advantageous than general anesthesia in obese patients, when applicable. Brachial nerve blocking is commonly used in upper extremity surgery and remains the first-line analgesic for shoulder surgeries.

Here, we report a case of severe dyspnea induced by HDP after supraclavicular block (SCB) in a morbidly obese patient, leading to conversion to general anesthesia with intubation, and review the relevant literature. Cases of phrenic nerve block with such severe consequences in morbidly obese patients are rarely reported. Written informed consent was obtained from the patient for the publication of clinical details and clinical images of the case report. This case report complies with the applicable EQUATOR publishing guidelines for case reports (CARE: Consensus-based Clinical Case Reporting).

## 2 Case report

A 67-year-old woman with a body mass index of 59.3 kg/m^2^ (height, 159 cm; weight, 150 kg) was admitted for open reduction and internal fixation of humeral shaft fracture. She had a history of type II diabetes mellitus, hypertension, cerebral infarction, and obstructive sleep apnea syndrome. On admission, her vital signs were stable, and her percutaneous oxygen saturation (SpO_2_) was 95% when 2 L/min of oxygen was administered via a nasal cannula. Physical examination revealed diminished breath sounds in the inferior portion of the left lung, without evidence of stridor or cardiac murmur. No deep vein thrombosis was found on preoperative ultrasound examination.

**Figure 1:**
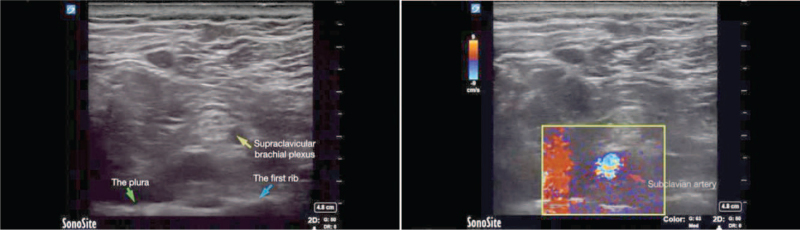
Supraclavicular brachial plexus scan in the patient with morbid obesity.

On airway assessment, the patient had a history of obstructive sleep apnea-hypopnea syndrome, neck circumference >47 cm, limitation of neck extension, interincisor distance >3 cm, thyromental distance >6 cm, and Mallampati class III, which suggested the possibility of difficult ventilation and intubation. After standard monitoring of cuff blood pressure, electrocardiography, and SpO_2_, we decided to administer BPB for anesthesia to avoid interference with the respiratory system and the risk of a difficult airway. We planned to perform an infraclavicular block (ICB) or costoclavicular block (CCB) to avoid the risk of a difficult airway related to general anesthesia and to minimize phrenic nerve involvement.^[3]^ However, the brachial plexus could not be displayed satisfactorily in the infraclavicular area after careful ultrasound inspection, and the brachial plexus in the costoclavicular space was located deeply (>5 cm deep by pressing the probe) with poor image quality even when using a convex transducer (1–5 Hz). Considering this and the anticipated steep needle trajectory angle, both of which may weaken the accuracy (effectiveness) and safety of the block, we subsequently opted for SCB, where the brachial plexus rested relatively shallow (∼4 cm deep by pressing the probe; see Figure 1 and Video S1, Supplemental Digital Content [Supplemental video. Supraclavicular brachial plexus scan in the patient with morbid obesity.] supraclavicular brachial plexus scan). The block was performed under the guidance of a linear transducer (4–15 Hz), with 12 mL of 0.33% ropivacaine injected and spread around the brachial plexus. Five minutes later, the patient developed restlessness and shortness of breath while SpO_2_ decreased to 82%. Noninvasive mask ventilation with 100% oxygen was applied immediately, but SpO_2_ could only be maintained at 92% to 93%, and the patient was intolerant to noninvasive mask ventilation. Auscultation revealed low breath sounds on the right side, with normal percussion notes. Immediate diaphragmatic ultrasound showed decreased diaphragmatic movement, suggesting the possibility of HDP. Further blood gas analysis indicated hypoxia (O_2_ partial pressure, 57 mm Hg) and hypercapnia (CO_2_ partial pressure, 79 mm Hg). Considering that the process of hypoxia and hypercapnia was undesirable in a patient with past cerebral infarction and prolonged convalescence for respiratory function, general anesthesia with video laryngeal scope-guided intubation was subsequently implemented to maintain respiratory function and vital signs (see Figure S1, Supplemental Digital Content general anesthesia with intratracheal intubation, and operation position).

The surgery lasted for 3 hours and was uneventful. The patient was then transferred to the intensive care unit (ICU). After excluding the presentation of pneumothorax and pulmonary embolism in the ICU, HDP was thought to be the main cause of dyspnea. Mechanical ventilation was required for four days until her respiratory function recovered. This led to a prolonged ventilation time (5 days) and increased length of ICU stay (5 days) and hospital stay (10 days).

## 3 Discussion

The phrenic nerve originates from the C3–C5 spinal nerves and runs on the surface of the anterior scalene muscle, initially in close proximity to the brachial plexus at the level of the cricoid cartilage and then descends over the anterior scalene into the thorax to innervate the diaphragm. When local anesthetic (LA) spreads to the phrenic nerve during BPBs, phrenic nerve palsy can occur, resulting in HDP. The reported incidence of HDP following different BPB approaches is summarized in Table 1. The incidence of HDP following interscalene block (ISB) is as high as 92% to 100%^[4]^ when administered with 20 mL LA, and could be 60% with 10 mL LA and 33% with 5 mL LA.^[5]^ The incidence of HDP during SCB is 70% with 25 mL LA and 34% to 67% with 20 mL LA.^[4,6]^ The incidence of HDP following different approaches of ICB is relatively low, which is 3% during the classical approach of ICB with 20 mL LA,^[6]^ 15% after retroclavicular block with 25 mL LA,^[7]^ and 2.5% to 5% after CCB (a new infraclavicular approach targeting the three cords that could provide sufficient analgesia for shoulder surgery by retrograde diffusion of LA to the supraclavicular nerve) with 20 to 30 mL LA, respectively.^[3,8]^ The combination of ICB and anterior suprascapular block for shoulder surgery yielded an HDP rate of 5%.^[9]^ The superior trunk block, a new block developed to minimize the involvement of the phrenic nerve by injecting LA next to the upper trunk of the brachial plexus (prior to the separation of the suprascapular nerve), has an HDP rate of 4.8%.^[10]^ Axillary tunnel catheter indwelling to avoid phrenic nerve block still resulted in a 1.5% incidence of HDP.^[11]^ HDP is a common complication of BPBs, as mentioned above, but it is usually well tolerated and may not show any symptoms in most patients. Thus, the risks and complications of HDP have been underestimated by many clinical practitioners.

**Table 1:** A summary of recent studies evaluating hemidiaphragmatic paralysis after ultrasound-guided branchial plexus blocks for shoulder and upper extremity surgery.

					Incidence of diaphragmatic paralysis			
Block type	Surgery	Injection dose	Control group	Analgesia effect	Experienmental	Control	Evidence level	Reference
Interscalene block	Shoulder arthroscopy	5 mL 0.75% ropivacaine	10 mL ISB	Similar to 10 mL ISB	33%	60%	Trial	Lee et al^[5]^
Superior trunk block	Shoulder arthroscopy	10 mL 0.5%bupivacaine	15 mL 0.5% bupivacaine ISB	Non- inferior to ISB	4.8%	71.4%	Trial	Kim et al^[10]^
Supraclavicular block	Shoulder arthroscopy	20 mL 0.375% Ropivacaine	20 mL 0.375% ropivacaine ISB	Similar to 20 mL ISB	58.3%	92%	Trial	Kim et al^[4]^
Retroclavicular block	upper extremity surgery	25 mL 0.5% Ropivacaine	25 mL 0.5% Ropivacaine SCB	Similar to SCB	15%	70%	Trial	Georgiadis et al^[7]^
Infraclavicular block	Upper extremity surgery	Varied in different studies	SCB	incomplete block in radial nerve higher in ICB	3%	34%	System review and meta-analysis	Park et al^[6]^
Infraclavicular subomohyoid block	Shoulder arthroscopy	20 mL 0.5% ropivacaine	5 mL 0.5% ropivacaine ISB	Similar to 5 mL ISB	5.6%	88.9%	Trial	Taha et al^[16]^
Costoclavicular block	Not mentioned	20–30 mL mixture of 1% lidocaine and 0.75% ropivacaine	SCB	NA	2.5%	39.8%	Retrospective analysis	Oh et al^[3]^
	Upper extremity surgery	20 mL mixture of 0.5% bupivacaine, 2% lidocaine and 1: 200 000 epinephrine	SCB	NA	5%	45%	Trial	Sivashanmugam et al^[8]^
Continuous axillary tunnel block^∗^	Shoulder and upper extremity surgery	30 mL 0.5% bupivacaine as loading dose; A bolus of 20 mL 0.25% bupivacaine infusion per 6 hours postoperatively	NA	NA	1.5%	NA	Evaluation Study	Cornish et al^[11]^
Combined axillary and anterior suprascapular nerve block	Shoulder arthroscopy	10 mL 0.375% Ropivacaine 2 mg dexamethasone	Combined axillary and posterior suprascapular nerve block	Posterior suprascapular method inferior to anterior suprascapular nerve block	7%(complete) 33%(Partial)	0%(complete) 2%(Partial)	Trial	Ferré et al^[17]^

CCB = costoclavicular block, HDP = hemidiaphragmatic paralysis, ICB = infraclavicular block, ISB = interscalene block, NA = not available, SCB = supraclavicular block.

∗ The authors of this article explained that they insert the catheter tip inside the axillary tunnel from supraclavicular region through a bent needle, which is considered as axillary tunnel block here.

The phrenic nerve is the only source of motor innervation of the diaphragm; therefore, it plays a crucial role in breathing. Although phrenic nerve block following BPB is usually asymptomatic in healthy patients,^[5,12]^ it may induce severe side effects in patients with compromised respiratory function, such as those with obstructive or restrictive ventilatory dysfunction, neuromuscular diseases, or morbid obesity. Obesity causes reduced functional residual capacity, a mismatched ventilation/perfusion ratio, and poor compliance. In obese patients, HDP can further reduce the forced vital capacity and forced expiratory volume in 1 second and increase the risk of dyspnea and hypoxic episodes.^[12]^ Moreover, the application of regional anesthesia in obese patients has other limitations, including a lower success rate and a higher incidence of complications.^[13]^ The anatomical landmarks in obese patients are not clear enough for peripheral blocks; therefore, an ultrasound-guided technique is recommended to increase the success rate. However, inadequate ultrasound visualization of target nerve structures still always occurs in obese patients, even when scanned by experienced clinical practitioners. Obesity also increases the risk of peripheral catheter-related infections under regional anesthesia.^[14]^

In the current case, both ICB and SCB provided sufficient anesthesia in the surgical region. We initially planned to perform ICB or CCB because of the relatively low reported incidence of HDP. Although CCB was reported to be more easily visualized than the traditional ICB approach^[3]^ because of the more superficial location of the brachial nerve cords and was reported as a successful alternative method for upper limb surgery in an obese patient,^[15]^ both methods failed due to inadequate visualization. Poor image quality and a large trajectory angle might affect the safety and effectiveness of regional anesthesia, resulting in malposition of the needle or catheter, leading to complications such as intravascular injection, intraneural injection, and pneumothorax. Consequently, we switched to SCB owing to its feasibility, which unfortunately led to severe respiratory dysfunction. To minimize the involvement of the phrenic nerve, new methods have been developed, such as superior trunk block, infraclavicular subomohyoid block, and CCB. However, no BPB approach could completely spare phrenic nerve involvement based on the available evidence. Therefore, clinicians should always consider the risk of HDP associated with BPB irrespective of the approach selected. BPBs should always be performed cautiously in patients with compromised respiratory functions.

In conclusion, we present a case in which an adult with morbid obesity experienced severe dyspnea after SCB due to unintentional phrenic nerve block and diaphragm paralysis. This case report aims to draw clinical practitioners’ attention to the risks and complications associated with BPBs. When performing BPBs, close monitoring of diaphragm movement with ultrasound, early recognition of HDP complications (e.g., emerging shortness of breath and decreased SpO_2_ decrease), and emergency strategies (e.g., immediate airway establishment) are important to prevent serious adverse consequences related to phrenic nerve block.

## Author contributions

**Conceptualization:** Jiaxin Lang, Xulei Cui.

**Investigation:** Jiaxin Lang.

**Resources:** Jia Zhang.

**Supervision:** Xulei Cui, Yuguang Huang.

**Writing – original draft:** Jiaxin Lang.

**Writing – review & editing:** Xulei Cui.

## Supplementary Material

Supplemental Digital Content

## Supplementary Material

Supplemental Digital Content
