# Supplementary material for: Dyspnea induced by hemidiaphragmatic paralysis after ultrasound-guided supraclavicular brachial plexus block in a morbidly obese patient
Source: Medicine (Baltimore). 2022 Jan 14;101(2):e28525. doi: 10.1097/MD.0000000000028525 (PMC8758049; doi:10.1097/MD.0000000000028525)
Supplement: Supplemental Digital Content [file medi-101-e28525-s002.doc]

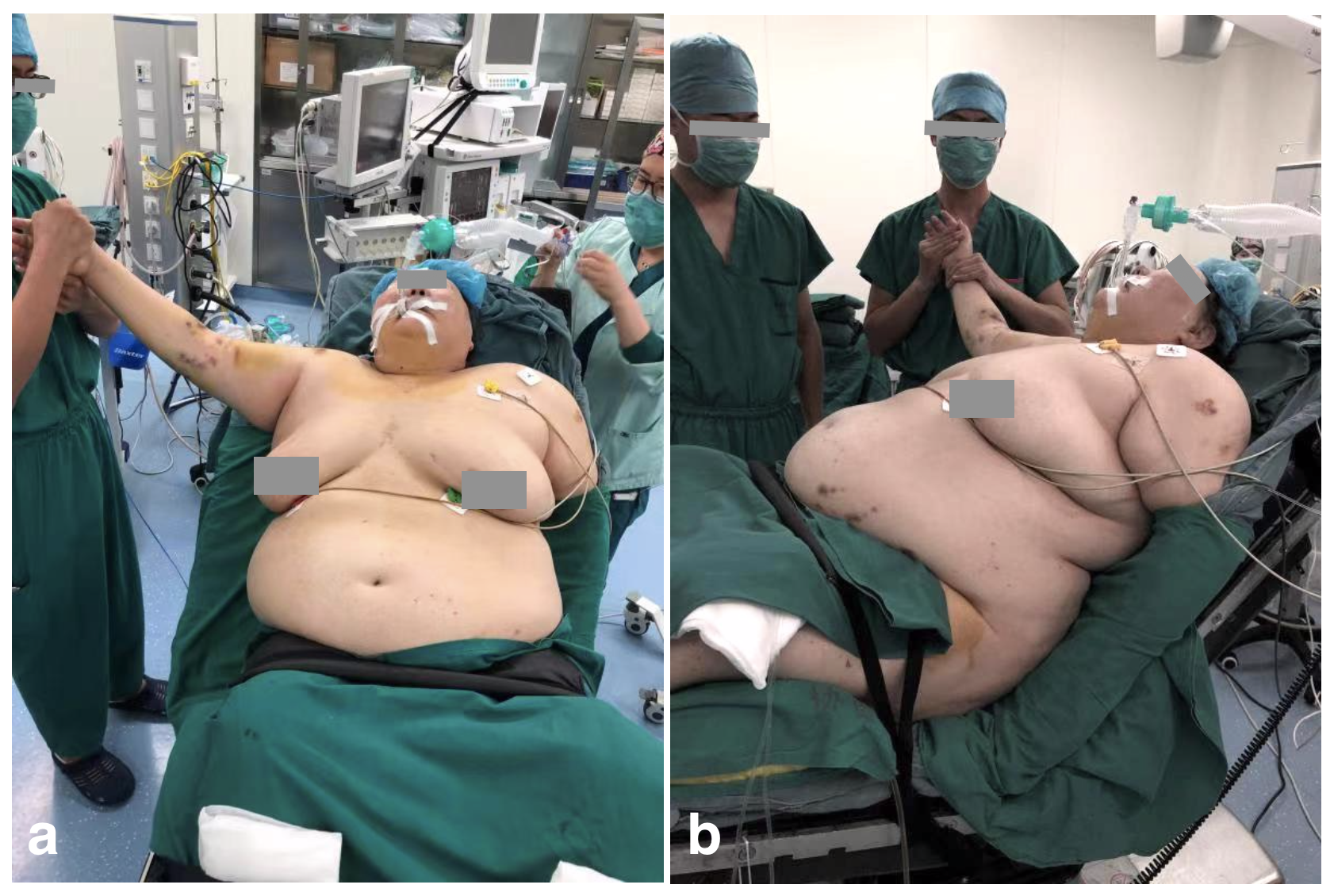


Supplemental Figure. General anesthesia with intratracheal intubation was performed in a mobidly obese patient following a supraclavicular block. a. Frontal image of the patient's operation positionb. Lateral image of the patient’s operation position.
